# Reclassification of type 2 diabetes to type 1 diabetes in Asturias (Spain) between 2011 and 2020

**DOI:** 10.1186/s13098-023-01069-y

**Published:** 2023-05-03

**Authors:** Raúl Rodríguez Escobedo, Carmen Lambert, Paula Morales Sánchez, Elías Delgado Álvarez, Edelmiro Menéndez Torre

**Affiliations:** 1 Servicio de Endocrinología y Nutrición. Hospitales Universitarios San Roque, Las Palmas de Gran Canaria, España; 2 Grupo de investigación en Endocrinología, Diabetes y Obesidad (ENDO), Instituto de Investigación del Principado de Asturias (ISPA), Oviedo, Asturias, España; 3 Universidad de Barcelona, Barcelona, España; 4 Centro de Investigación Biomédica en Red en Enfermedades Raras (CIBERER), Madrid, España; 5 Servicio de Endocrinología y Nutrición. Hospital Universitario Central de Asturias. Oviedo, Asturias, España; 6 Departamento de Medicina, Universidad de Oviedo. Oviedo, Asturias, España

**Keywords:** Type 1 diabetes, Type 2 diabetes, Diagnosis, Insulin, Epidemiolgy, Reclassification, LADA

## Abstract

**Background:**

Differentiating between type 1 diabetes (T1D) and type 2 diabetes (T2D) can be difficult in adults. The aim of this study was to determine the frequency of diagnostic reclassification from T2D to T1D, the characteristics of the patients and the impact on the management of the disease.

**Methods:**

Observational and descriptive study including patients diagnosed with T1D in Asturias (Spain) between 2011 and 2020 who had been considered as T2D for at least 12 months.

**Results:**

A total of 205 patients were included, representing 45.3% of those diagnosed with T1D over 30 years of age. Median time of evolution as T2D was 7,8 years. The age was 59.1 ± 12.9 years. BMI was > 25 kg/m^2^ in 46.8% of patients. HbA1c was 9.1 ± 2.1%, 77 ± 22 mmol/mol, and 56.5% were using insulin. Pancreatic antibodies were present in 95.5%, the most frequent being GAD, 82.6%. At 6 months, basal insulin use increased from 46.9 to 86.3%, and HbA1c decreased, 9.2 ± 2.0%vs7.7 ± 1.2%, 77 ± 22vs60 ± 13 mmol/mol; p < 0.0001.

**Conclusions:**

Diagnosis as T2D in patients with T1D in adults is common. Age, BMI, insulin use and other clinical features are not definitely discriminatory. GAD is the antibody of choice in case of diagnostic suspect. Reclassification has important implications for metabolic control.

## Introduction

Type 1 diabetes (T1D) and type 2 diabetes (T2D) are two similar diseases characterized by hyperglycemia, but with important pathophysiological differences between them. T1D is distinguished by an autoimmune beta-cell destruction, leading to an absolute insulin deficiency, and therefore, requiring an exogen insulin treatment. In contrast, in T2D self-production of insulin is maintained and a response to non-insulin therapy can be obtained [1].

Despite these differences, distinguishing between T1D and T2D can be sometimes difficult, especially in adults [2]. In fact, around 40% of patients with T1D onset over the age of 30 years are initially diagnosed as T2D [3, 4]. The rate of misdiagnosis increases with the age of onset of diabetes [3]. This is especially relevant considering that 40% of new cases of T1D occur in those over 30 years of age [5]. In a review published in 2021, there are listed the top 5 causes why people with T1D were misdiagnosed as T2D [6]: (1) lack of awareness that the onset of type 1 diabetes is not limited to children; (2) the overwhelming majority of people developing diabetes as older adults have type 2 diabetes, contributing to a confirmation bias; (3) typical clinical criteria, such as body mass index (BMI) and metabolic syndrome, can be poor discriminators, especially as rates of obesity in the overall population increase; (4) clinical characteristics of adult-onset type 1 diabetes can masquerade as type 2 diabetes and (5) lack of awareness of and accessibility to biomarkers that may serve as tools to distinguish type 1 diabetes and type 2 diabetes.

In this situation, it is recommended to maintain a high level of suspicion for T1D, particularly in patients younger than 35 years, BMI under 25 kg/m^2^, personal history of autoimmune diseases, family history of T1D or other autoimmune diseases and/or lack of metabolic control with non-insulin treatments [7, 8]. The most discriminative clinical characteristics for diagnosis are age, time to insulin and BMI, however, none is perfect [9]. Age is the most discriminating element, but it leads to the error of identifying T1D as early age and T2D as late age. The need for insulin in the first 3 years of the disease is suggestive of T1D [4, 7] but time to insulin is in many cases dependent on the attending healthcare professional or on the patient’s own wishes. Regarding BMI, majority of older adults with low BMI will have T2D [10].

In 2021, a consensus was published by the American Diabetes Association (ADA) and the European Association for the Study of Diabetes (EADS) on the management of T1D in adults [8]. This consensus recommends the assessment of pancreatic autoimmunity in all patients with a suspected diagnosis of T1D. Considering that the presence of one or more pancreatic antibodies is highly predictive of rapid progression to severe insulin deficiency [11, 12], a diagnosis of T1D is recommended, even in those patients who do not yet require the use of insulin. In case of negative antibodies, assessment of age, C-peptide, and other data such as BMI, weight loss or ketoacidosis is recommended.

An accurate diagnosis from the beginning is essential for the management of the disease. In T1D, knowledge of the disease and the need to adapt one’s lifestyle is essential for good control of the disease, therefore, the initial diagnosis as T2D produces an increase in the patient’s confusion in a situation that already it is stressful in itself [6, 13]. In addition, the treatment received by the patient may not be adequate; in fact, 38% of patients with adult-onset T1D do not receive insulin at the time of diagnosis [4]. Thus, T1D misdiagnosis is correlated with diabetes ketoacidosis [6]. It is important to emphasize that the therapeutic management of both diseases is different [14], and that those drugs commonly used in T2D may not be useful in T1D or even increase the risk of complications such as diabetic ketoacidosis that is increased with the use of sodium-glucose cotransporter type 2 inhibitors (iSGLT2) [15], especially if the insulinopenia status is unknown. Similarly, for other health actions such as hospitalizations for other causes, it is essential to know the diagnosis of T1D to avoid treatment errors such as withholding insulin [3]. Furthermore, without a diagnosis of T1D, the patient has no opportunity to benefit from interventions restricted to this disease, such as glucose monitoring or the use of insulin infusers [4]. Thus, correct diagnostic classification is crucial to achieve appropriate disease management.

## Methods

The aim of this study was to determine the frequency of diagnostic reclassification from T2D to T1D in Asturias and to define the characteristics of this group of patients. In addition, we have studied the impact of reclassification on the treatment and on metabolic control of these patients. The final objective was to raise awareness of this situation and facilitate the identification of these patients to improve the management of the disease.

An observational and descriptive study was conducted in which new diagnoses of T1D between 2011 and 2020 in Asturias that had been previously diagnosed with T2D for at least 12 months were included. Data were obtained from the T1D incidence study conducted in the region [16]. Diabetes mellitus was defined as meeting the criteria for the diagnosis of diabetes mellitus established by the American Diabetes Association (ADA) [1]. Diagnosis of T1D was based on the ADA and EASD consensus guidelines for the management of T1D in adults [8]. Therefore, islet autoantibodies was considered: glutamic acid decarboxylase (GAD), islet tyrosine phosphatase2 (IA2) and zinc transporter 8 (ZNT8), all of them measured with the RSR ELISA assay (RSR, Cardiff, U.K.). Islet autoantibodies were considered positive if GAD > 10 units/ml, IA2 > 10 units/ml and ZNT8 > 20 units/ml, according to the reference values of our reference laboratory. The study did not change the usual clinical practice, so autoimmunity testing was only performed in patients with clinical features, that aroused suspicion of type 1 diabetes mellitus. In cases of undetermined or negative antibodies, clinical characteristics such as age at onset of diabetes, C-peptide, BMI and insulin use were taken into consideration, as well as the criteria of attending healthcare professional.

We analyzed the frequency of reclassification with respect to the total number of patients diagnosed with T1D in Asturias in the years of the study. Not only clinical characteristics and, but also the anti-diabetic, lipid-lowering and antihypertensive treatment prescribed for each patient at the time of reclassification and six months after this, as well as the glycosylated hemoglobin (HbA1c) at both times, were collected for this study by reviewing medical records.

Statistical differences were analysed by Wilcoxon test. The statistical analysis software used for this calculation was R (R Development Core Team), version 4.1.3.

The study has been approved by the Research Ethics Committee of the Principality of Asturias, project number 2020.323.

## Results

**Fig. 1:**
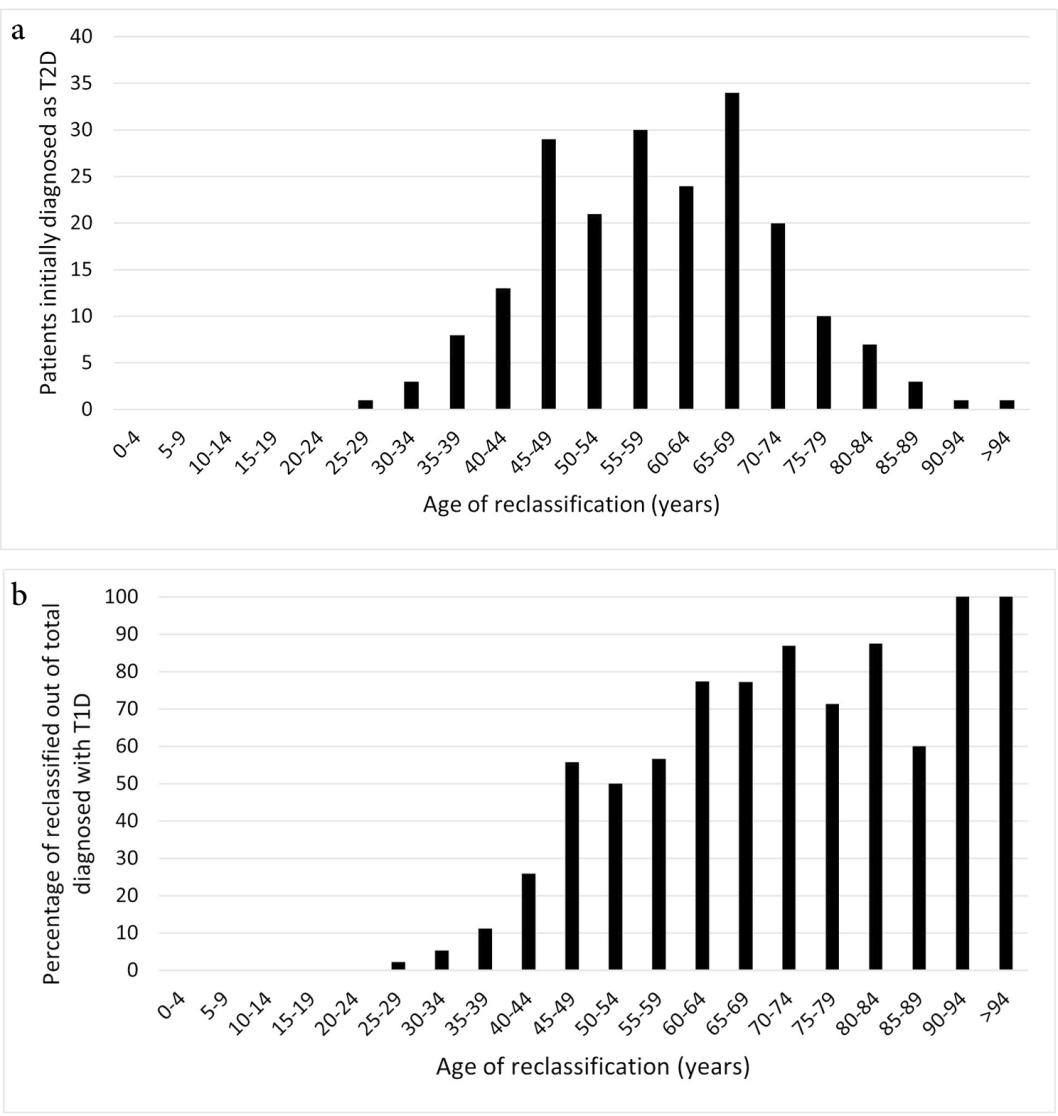
Patients according to age diagnosed as T1D at least 12 months with diagnosis of T2D according to **(a)** Number of patients and **(b)** Percentage with respect to total diagnosed with T1D.

We found 205 patients, 100 men and 105 women, diagnosed with T1D after having been considered for at least 12 months as T2D. The mean age of the patients was 59.1 ± 12.9 years. The distribution by age group is shown in Fig. 1a. Patient characteristics are given in Table 1. The percentage of patients over 30 years of age in whom diagnostic reclassification was performed with respect to the total diagnoses of T1D was 45.2%; the percentage of misdiagnosed patients grouped by age is shown in Fig. 1b.

**Table 1:** Characteristics of patients diagnosed as T1D after at least 12 months as T2D and of patients directly diagnosed as T1D.

	Male	Female	Total	Total directly diagnosed as T1D
Patients (n)	100	105	205	247
Age (years)	56.2 ± 12.3	61.9 ± 12.9	59.1 ± 12.9	44.3 ± 12.0
Evolution time (years median, IQ25%-75%)	9.6 (3.9–15.8)	6.4 (2.5–13.4)	7.8 (2.9–14.8)	-
BMI (kg/m^2^)	25.6 ± 3.8	25.7 ± 5.9	25.7 ± 5.0	24.6 ± 5.7
< 25 (%)	49.3	56.6	53.2	60.5
25–30 (%)	38.7	24.1	31.0	26.2
> 30 (%)	12	19.3	15.8	13.3
HbA1c (%, mmol/mol)	9.1 ± 2.1, 76 ± 23	9.2 ± 2.0, 77 ± 22	9.1 ± 2.1, 77 ± 22	11.0 ± 2.8
Insulin use (%)	39.7	70.9	56.5	-
Autoimmune thyroid disease (%)	17.2	29.1	23.3	14.4
Hypothyroidism (%)	12.1	21.5	16.8	11.5
Hyperthyroidism (%)	3.0	6.8	4.9	2.9
Family history of T1D (%)	14.5	11.0	12.8	12.4
Pancreatic autoimmunity (%)	96.9	94.2	95.5	94.0
GAD (%)	81.6	83.5	82.6	84.4
IA2 (%)	23.2	36.9	30.3	42.1
ZNT8 (%)	26.5	30.5	28.7	50.0
C-peptide (ng/ml; median, IQ25%-75%)	0.88 (0.27–1.39)	0.88 (0.28–1.56)	0.88 (0.28–1.45)	1.15 (0.01-11.0)

**Fig. 2:**
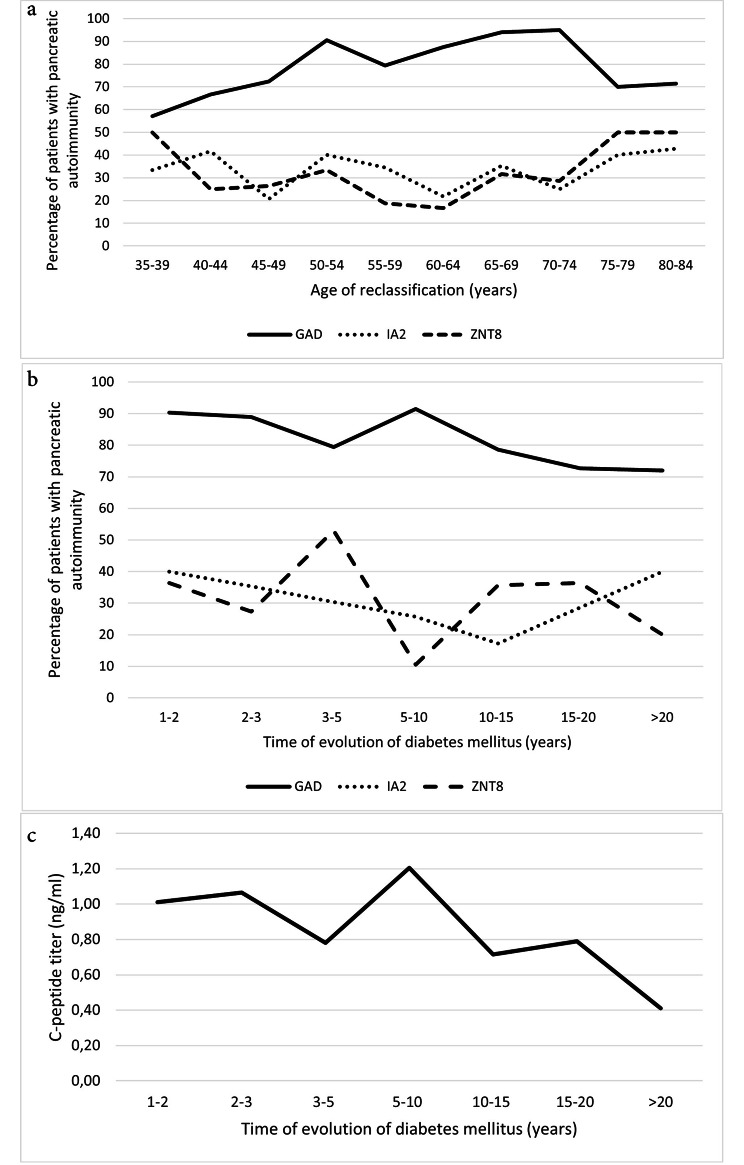
**(a)** Percentage of patients with GAD, IA2 and ZNT8 autoimmunity by age group. **(b)** Percentage of patients with GAD, IA2 and Zn autoimmunity and **(c)** C-peptide titer according to time of evolution of diabetes mellitus

In 95.5% of the patients, the pancreatic antibodies studied were positive for at least one of the antibodies studied. GAD positivity was found in 82.6% of patients, IA2 in 30.3% and ZNT8 in 28.7%. The distribution of autoimmunity by age group is shown in Fig. 2a. The distribution of pancreatic autoimmunity and C-peptide positivity as a function of the time course of diabetes mellitus is shown in Fig. 2b and c.

Prior to reclassification as T1D the most used treatment was metformin, indicated in 70.8% of cases, followed by basal insulin, 46.9%, and iDPP4, 46.3%. Six months after diagnosis, the most employed treatment was basal insulin, 86.3%, followed by rapid insulin 52.5%, with metformin in third place, 33.1%. Changes in treatment are shown in Table 2.

**Table 2:** Percentage of drug use at the time of diagnostic reclassification and at 6 months

	Basal	6 months	p
Basal insulin	46.9%	86.3%	< 0.0001
Bolus Insulin	19.7%	52.5%	< 0.0001
Premixed insulin	13.6%	9.4%	0.261
Metformin	70.8%	33.1%	< 0.0001
iDPP4	46.3%	23.0%	< 0.0001
aGLP1	3.4%	2.2%	0.524
iSGLT2	10.9%	7.2%	0.278
Sulfonylurea	8.8%	1.4%	0.005
Glinides	8.2%	1.4%	0.008
Pioglitazone	0%	0%	-
Statin	37.4%	41.7%	0.456
Ezetimibe	0.7%	2.2%	0.287
Antihypertensive	22.5%	25.2%	0.588

**Fig. 3:**
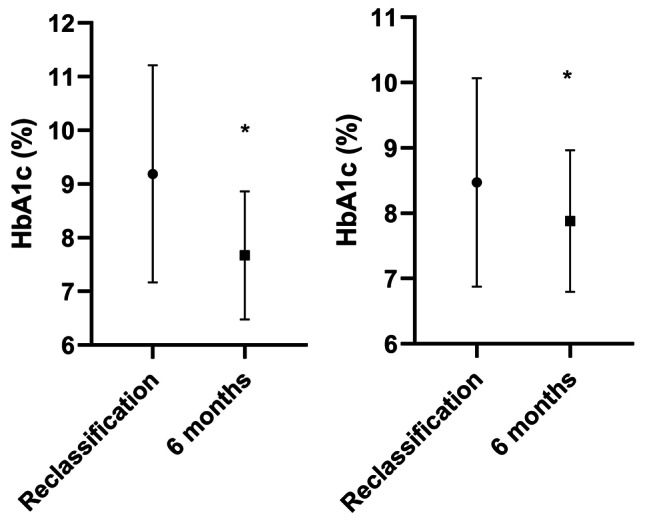
HbA1c at the time of reclassification and at six months **(a)** in the sample as a whole, 9.2 ± 2.0 to 7.7 ± 1.2%, from 77 ± 22 to 60 ± 13 mmol/mol (p < 0.0001), and **(b)** in those patients already treated with insulin in basal-bolus insulin therapy, 8.5 ± 1.6 vs. 7.9 ± 1.1%, 69 ± 18 vs. 63 ± 12 mmol/mol (p = 0.0081). Expressed as mean ± SD.

There was a reduction in HbA1c from 9.2 ± 2.0 to 7.7 ± 1.2%, from 77 ± 22 to 60 ± 13 mmol/mol, p < 0.0001; expressed in Fig. 3a. The greatest decrease in HbA1c was obtained in those patients who were started on basal insulin, with a 2.3%, 24 mmol/mol, reduction, 9.7 ± 1.9 vs. 7.4 ± 1.1%, 82 ± 21 vs. 58 ± 13 mmol/mol, p < 0.0001. The addition of rapid insulin resulted in a 1.9%, 22 mmol/mol decrease in HbA1c, 9.5 ± 2.0 vs. 7.6 ± 1.1%, 81 ± 21 vs. 59 ± 12 mmol/mol, p < 0.0001.

There were 22 patients who, prior to reclassification, were being treated with basal-bolus insulin therapy. In 18.18% of them, non-insulin antidiabetic treatment was eliminated. HbA1c evolution showed a reduction in HbA1c, 8.5 ± 1.6 vs. 7.9 ± 1.1%, 69 ± 18 vs. 63 ± 12 mmol/mol (p = 0.0081), as shown in Fig. 3b.

## Discussion

Our study shows that 45.2% of diagnoses of T1D in patients over 30 years of age had previously been considered as T2D. This percentage is higher than that reflected in other studies, which show a frequency of around 40% [3, 4], even though in our study a diagnostic adjustment interval of 12 months has been considered. The time of evolution with an inaccurate diagnosis is very high, with a median of almost 8 years. Therefore, misdiagnosis is frequent and this misclassification lasts over time. A wrong diagnosis of the disease, as already mentioned, has important repercussions on the health care received by the patient.

Most cases of reclassification have been performed in patients between 45 and 70 years, been the age range with the highest number of patients being 65–69 years. However, analysis of the frequency of diagnostic reclassification according to the incidence of T1D shows that it increases with age. Other studies have shown the same situation [3], which is explained by the higher level of suspicion of T1D occurring at younger ages. This shows that age is not a fully discriminatory element and that the suspicion of T1D only in those under 35 years of age increases the risk of misdiagnosis of the disease.

BMI assessment showed that more than half of the patients were under 25 kg/m^2^, which increases the level of suspicion of T1D. However, almost a third had overweight and 15.8% had obesity. Once again, the clinical characteristic may help in the diagnostic distinction but it can also favor the error in patients with characteristics that are far from the usual profile of T1D. This is particularly relevant if we consider the increase in overweight and obesity in the general population [17], which leads to an increase in T1D diagnoses in these BMI ranges.

Our patients had poor metabolic control, with a mean HbA1c above 9%. As mentioned, the difficulty in glycemic control is another element of suspicion of a possible misdiagnosis [6]. Additionally, the early need for insulin use is another indicative of T1D [9]. In our population, almost half of the patients did not use insulin, especially in males. The fact that patients with poor metabolic control did not receive insulin treatment shows the variability that may exist in insulin use.

The association between autoimmune thyroid disease and T1D is widely described [18, 19]. In our study almost a quarter of the patients had thyroid autoimmunity, mainly in women. Therefore, we found another feature that invites us to reconsider the diagnosis. There is also a high frequency of family history of T1D.

Once the diagnostic suspicion has been established, the main analytical test recommended is pancreatic autoimmunity [8]. Our study shows that, almost practically all patients were positive for any of the autoimmune markers studied. Specifically, GAD showed the highest positivity, with a frequency of 82.6%. In fact, GAD is the antibody that has shown higher frequencies and, in addition, a greater capacity to maintain positivity over time [20, 21]. Therefore, in the case of a suspected diagnosis of T1D, it is recommended to begin the study with the evaluation of GAD, leaving the rest of the antibodies for analysis in the case of negativity of GAD [8]. In this way, in addition to the diagnosis of T1D, an optimization of resources is achieved. Other studies show that the determination of pancreatic autoimmunity is useful not only for the distinction between T1D and T2D, but also for the management of patients with T1D. In fact, one study shows that successful withdrawal of insulin treatment was possible in 22.6% of patients with T1D and negative autoimmunity [22]. However, the determination of pancreatic autoimmunity is only recommended in those patients with clinical suspicion of T1D and not in all patients with diabetes as it could lead to false positives. In this sense, interpretation with caution is recommended, remembering that “the presence of a biomarker that can occur in the absence of disease should not define a disease state” [23].

Latent Autoimmune Diabetes of Adults (LADA) [24], referred as “Slowly evolving immune-mediated diabetes” by the WHO [25], is a controversial concept classically defined as diabetes mellitus in patients older than 35 years, with clinical features compatible with T2D and positive autoimmunity. It is interesting to note that in the ADA and EASD consensus on T1D in adults [8] in which the differentiation with other types of diabetes mellitus is discussed, reference is made only once to LADA. Specifically, to indicate that it is still under discussion whether it is a milder form of T1D or a mixture of patients with T1D or T2D. In this regard, the annual ADA guidelines include all forms of diabetes mediated by autoimmune destruction of the beta-cell under the diagnosis of T1D [1]. Taking this into account in this study the term LADA is abandoned and referred to as T1D.

The recommended treatment in T1D and T2D is different, so therapeutic changes are expected after reclassification [14]. Indeed, our study shows an increase in the use of basal insulin, as corresponds to the usual management of T1D, as well as a decrease in the use of non-insulin antidiabetic drugs. The high use of non-insulin antidiabetic drugs may be surprising, but it is justified by the fact that at 6 months the full process of therapeutic change may not yet have been completed. In this sense, a slight increase in the use of lipid-lowering and antihypertensive drugs is observed, probably because of increased attention to cardiovascular disease risk factors in these patients.

At 6 months after diagnostic reclassification, a statistically significant improvement in Hb1c was observed. This shows that the adaptation of treatment to T1D achieves an improvement in metabolic control. However, it is worth noting that patients who were already using basal-bolus insulin therapy for T2D also significantly improved their HbA1c, even when non-insulin antidiabetic drugs were withdrawn. This demonstrates the importance of a correct diagnosis even beyond pharmacological treatment. Diagnostic reclassification to T1D involves a different management, with special attention to diabetes education and therefore greater knowledge of the disease, which has an impact on improving metabolic control and on reducing of potential risk of diabetic ketoacidosis.

The main limitation of this study is that only patients with a suspected diagnosis of subsequently confirmed T1D have been included, however, it is possible that many patients with T1D are still considered to have T2D so it is not possible to know the true magnitude of the misdiagnosis. Another limitation is that it has not been possible to compare the characteristics of our patients with those in whom the diagnostic suspicion of T1D was raised but the classification as T2D was finally maintained. The main strength of the study is that it exposes a reality that directly affects healthcare. It shows the high frequency of misdiagnosis, presents the characteristics of these patients to facilitate their identification and points out the main test to study the diagnostic suspicion. Furthermore, we have not found other studies like ours that analyze the evolution of treatment and metabolic control once reclassification has been carried out; also showing its importance in this regard.

## Conclusion

The diagnosis as T2D in cases of T1D is frequent in adults and is sustained over a long period of time. Clinical data such as age, BMI, metabolic control, insulin use, personal history of autoimmune diseases or family history of T1D may serve as elements of suspicion, but none of them is fully discriminative. Pancreatic autoimmunity is fundamental in the diagnosis of T1D and it is recommended to initiate the study with GAD given its higher prevalence. Diagnostic reclassification implies changes in the patient management that results in an improved metabolic control. Ultimately, this study shows the importance of maintaining a high level of suspicion in the care of patients initially classified as T2D given the possibility of diagnostic error, which has a negative impact on the management of the disease.

## Data Availability

The data used during the current study are available from the corresponding author on reasonable request.

## Abbreviations

T1D: Type 1 diabetes
T2D: Type 2 diabetes
BMI: Body mass index
ADA: American Diabetes Association
EADS: European Association for the Study of Diabetes
iSGLT2: Sodium-glucose cotransporter type 2 inhibitors
GAD: Glutamic acid decarboxylase
IA2: Islet tyrosine phosphatase2
ZNT8: Zinc transporter 8
HbA1c: Glycosylated hemoglobin
LADA: Latent Autoimmune Diabetes of Adults
